# Anti-Inflammatory Effects of Bangpungtongsung-San, a Traditional Herbal Prescription

**DOI:** 10.1155/2012/892943

**Published:** 2012-07-29

**Authors:** Chul Won Lee, Sang Chan Kim, Tae Won Kwak, Jong Rok Lee, Mi Jeong Jo, Yong-Tae Ahn, Jong Myoung Kim, Won G. An

**Affiliations:** ^1^Institute of Marine BioTechnology, Pusan National University, Busan 609-735, Republic of Korea; ^2^College of Oriental Medicine, Daegu Haany University, Daegu 706-060, Republic of Korea; ^3^Division of Pharmacology, School of Korean Medicine, Pusan National University, Yangsan 626-870, Republic of Korea; ^4^Department of Marine BioMaterials and Aquaculture, Pukyong National University, Busan 608-737, Republic of Korea

## Abstract

Bangpungtongsung-san (BPTS), a traditional oriental herbal prescription, is widely used for expelling wind, draining heat, and providing general improvement to the immune system. In this study, we investigated the effects of BPTS on induction of inducible nitric oxide synthase (iNOS), cyclooxygenase-2 (COX-2), proinflammatory cytokines, nuclear factor-kappa B (NF-**κ**B), and mitogen-activated protein kinases (MAPKs) in lipopolysaccharide- (LPS- ) stimulated Raw 264.7 cells, and on paw edema in rats. At concentrations of 0.5, 0.75, and 1 mg/mL, treatment with BPTS inhibited levels of expression of LPS-induced NF-**κ**B and MAPKs (ERK, JNK, and p38) as well as production of proinflammatory mediators, such as nitric oxide (NO), prostaglandin E_2_ (PGE_2_), tumor necrosis factor-**α** (TNF-**α**), and interleukin-6 (IL-6) by LPS. These results suggest that BPTS may exert anti-inflammatory effects via reduction of proinflammatory mediators, including NO, PGE_2_, TNF-**α**, and IL-6 through suppression of the signaling pathways of NF-**κ**B and MAPKs in LPS-induced macrophages. In addition, using the carrageenan-induced paw edema assay, an antiedema effect of BPTS was observed in rats. These findings may provide scientific evidence validating the use of BPTS in treatment of patients with heat syndrome in Korean oriental medicine.

## 1. Introduction

BPTS is described in Xuan Ming Lun Fang, written by Wansu Liu in A.D. 1172 [1]. BPTS is a traditional herbal prescription for treatment of both exterior and interior syndromes. In general, BPTS has been used for relief of pathogenic wind-heat and control of excessive exterior and interior syndromes marked by cold fit, high fever, headache, vertigo, bitter taste, dry throat, constipation, yellow and greasy coating of the tongue, rapid or slippery pulse, and scanty yellow urine [2].

According to oriental medicine in Korea, the conditions of disease have been classified into three types, that is, deficiency syndrome, cold syndrome, and heat syndrome. Use of Yieum-jeon [3],  Ojeok-san [4],  and BPTS for treatment of one of these three types of disease, respectively, has been reported [2]. Heat syndrome is caused mainly by pathogenic heat or hyperactivity of *yang-qi*, usually seen with infectious disease. Thus, BPTS can be regarded as having a therapeutic effect against inflammatory disease.

Inflammation, the first response of the immune system to infection or irritation, is the defensive reaction in helping a body fight disease. This response causes progressive damage in the long term; therefore, it should be mitigated. Multiple inflammatory factors are involved in inflammatory reaction. Among them, NO, PGE_2_, and cytokines, such as TNF-*α* and IL-6, play pivotal roles [5].  NO is a free radical produced from L-arginine by nitric oxide synthases (NOS) [6]. Under normal physiological conditions, NO production has beneficial microbicidal, antiviral, antiparasitic, and antitumoral effects. However, inordinate NO generated by iNOS is a mediator of a variety of inflammatory diseases [7, 8]. In addition, the enzyme involved in production of prostaglandins, such as PGE_2_, is cyclooxygenase (COX). In particular, COX-2 is the enzyme responsible for mediating inflammation through production of PGE_2_  induced by LPS, and so forth [9, 10]. TNF-*α* is known to play a key role in many autoimmune diseases. In addition, it is another primary mediator in the inflammatory reaction that causes innate immune responses by stimulating secretion of other inflammatory cytokines [11, 12]. IL-6 plays a role in the acute response and induces an increase in antibody production through activation of lymphocytes. The level of this cytokine has been reported to always show an increase in inflammatory lesions [12, 13].

NF-*κ*B is one of the most important transcription factors involved in transactivation of a variety of genes associated with regulation of immune and inflammatory responses, cellular proliferation, and tumorigenesis [14]. In response to LPS, it controls expression of genes for iNOS, COX-2, and many proinflammatory cytokines. Inappropriate activation of NF-*κ*B shows correlation with a variety of inflammatory diseases. Many anti-inflammatory drugs inhibit production of proinflammatory cytokines via suppressing activation of NF-*κ*B [15]. Thus, suppressors that control activation of NF-*κ*B play an important role in treatment of inflammatory diseases. 

Mitogen-activated protein kinases (MAPKs) transduce extracellular signals into a variety of cellular processes, including cell differentiation, proliferation, survival, and apoptosis [16, 17]. They are comprised of three principal family members (extracellular signal-regulated kinases (ERKs), c-Jun NH_2_-terminal kinases (JNKs), and p38) and are a group of signaling molecules that appear to play important roles in inflammatory processes and development of cancer [17–21]. The MAPKs-dependent signaling pathway activates NF-*κ*B and induces expression of proinflammatory genes [22–25].

Findings from several studies have demonstrated that BPTS can induce a decrease in body weight through reduction of total lipid in serum [26]; it has also been reported to reduce hypertension [27]. In addition, BPTS exhibits antihistamine and antiallergic effects [28]. However, no studies of the effects of BPTS on inflammation have been reported. In this study, we examined the effects of BPTS as an anti-inflammatory prescription on NF-*κ*B and MAPKs dependent induction of NO, PGE_2_, and proinflammatory cytokines in Raw 264.7 cells stimulated with LPS. In addition, we performed a paw edema assay to evaluate the effect of BPTS on acute phase inflammation in an animal model. Through these processes, we were able to verify the anti-inflammatory effects of BPTS.

## 2. Materials and Methods

### 2.1. Chemicals and Reagents

Five reference standards, paeoniflorin, glycyrrhizin, ephedrine, nodakenin, and wogonin, were purchased from Wako Inc. (Japan). Purity of the five reference standards was greater than 98%. HPLC grade solution, acetonitrile, methanol, and reagents were purchased from J.  T.  Baker (USA). Anti-iNOS and Anti-*β*-actin antibodies were purchased from Calbiochem (San Diego, CA, USA). Anti-COX-2, anti-NF-*κ*B p65, anti-I*κ*B*α*, and anti-lamin A/C antibodies were obtained from Santa Cruz Biotechnology Inc. (Santa Cruz, CA, USA), and ERK1/2, phospho-ERK1/2, JNK, phospho-JNK, p38, and phospho-p38 antibodies were purchased from Cell Signaling Technology, Inc. (Danvers, MA, USA); peroxidase-conjugated secondary antibody was purchased from Santa Cruz Biotechnology Inc.. The enzyme immunoassay kit for PGE_2_ was purchased from R&D Systems (Minneapolis, MN, USA), and the TNF-*α* and IL-6 ELISA Kits were obtained from Pierce Endogen (Rockford, IL, USA). The luciferase assay system was purchased from Promega (Madison, CA, USA). 3-(4,5-dimethylthiazol-2-yl)-2,5-diphenyl tetrazolium bromide (MTT), sulfanilamide, lipopolysaccharide (LPS), carrageenan, dexamethasone, and all other chemicals were purchased from the Sigma Aldrich Chemical Co. (St. Louis, MO, USA).

### 2.2. Preparation of BPTS

BPTS prescription (41.8 g) included the following dried herbal medicines: 4.8 g of *Glycyrrhizae radix*, 2.8 g of *Gypsum fibrosum*, 2.8 g of *Scutellariae radix*, 2.8 g of *Platycodi radix*, 1.8 g of *Ledebouriellae radix*, 1.8 g of *Cnidii rhizoma*, 1.8 g of *Angelica gigas radix*, 1.8 g of *Paeoniae radix rubra*, 1.8 g of *Rhei rhizoma*, 1.8 g of *Ephedrae herba*, 1.8 g of *Menthae herba*, 1.8 g of *Forsythiae fructus*, 1.8 g of *Natrii sulfas*, 1.4 g of *Schizonepetae spica*, 1.4 g of *Atractylodis rhizoma alba*,  1.4 g of *Gardeniae fructus*, 1.4 g of *Zingiberis rhizoma crudus*, and 6.8 g of talcum. These herbal medicines were obtained from the School of Korean Medicine, Pusan National University, Korea. Voucher specimens (PNU10-10) have been deposited into the Herbarium of Ducom. Following extraction of the herbal mixture (41.8 g) with 800 mL of boiling distilled water for 3 h, it was filtered through a filter paper (Hyundai Micro No. 20). The supernatant was filtered through a 0.2 *μ*m filter (Nalgene, New York, USA), followed by lyophilization of the filtrate. A yield of 19.5% was obtained from the lyophilized BPTS extract. BPTS powder was dissolved in distilled water prior to use.

### 2.3. Profiling the Chemical Contents of BPTS by UPLC

#### 2.3.1. Chromatography Conditions

The UPLC (ultra performance liquid chromatography) system (Waters, USA), which was equipped with a pump Waters ACQUITY ultra performance LC system (USA) and a Waters ACQUITY photodiode array detector (PDA), was used for analysis. The empower data system was used for recording of the output signal of the detector. Separation was executed on a Waters ACQUITY BEH C_18_ column (1.7 *μ*m, 2.1 × 100). The mobile phase was composed of water and acetonitrile with the gradient elution system at a flow rate of 0.4 mL/min. The injection volume was 2 *μ*L. The detection UV wavelength was set at 230, 254, and 330 nm. The column temperature was set at room temperature.

#### 2.3.2. Preparation of Standard Solutions and Sample

Standard stock solutions of five marker components, paeoniflorin, glycyrrhizin, ephedrine, nodakenin, and wogonin, were prepared by dissolving at a concentration of 100 *μ*g/mL in 10 mL of methanol. Standard stock solutions were diluted with methanol for production of working standard solutions. Standard stock solutions and working solutions were stored at 4°C. 

### 2.4. Cell Culture

Raw 264.7 mouse macrophage cells (American Type Culture Collection) were maintained in Dulbecco's modified Eagle's medium (DMEM; Hyclone, Thermo Scientific Inc., Bremen, Germany) supplemented with 10% heat-inactivated fetal bovine serum (FBS; Sigma, St. Louis, MO, USA), 100 U/mL of penicillin, and 100 *μ*g/mL of streptomycin (Gibco/BRL, Grand Island, NY, USA) at 37°C in a 5% CO_2_ incubator. 

### 2.5. MTT Assay for Cell Viability

For determination of cytotoxic concentrations of BPTS, Raw 264.7 cells were plated at a density of 5 × 10^4^ cells per well in a 96-well plate. Cells were serum-starved for 16 h and pretreated with a variety of concentrations of BPTS for 1 h, followed by stimulation with 1 *μ*g/mL of LPS. Cells were then incubated for the next 20 h at 37°C, 5% CO_2_ incubator. Following incubation of the cells, viable cells were stained with 3-(4,5-dimethylthiazol-2-yl)-2,5-diphenyltetrazolium bromide (0.5 mg/mL, 4 h). Media were then removed and formazan crystals produced in the wells were dissolved by addition of 200 *μ*L dimethylsulfoxide. Absorbance was measured at 570 nm using an ELISA microplate reader (Tecan, USA). Cell viability was defined relative to untreated control cells (i.e., viability (% control) = 100 × (absorbance of treated sample)/(absorbance of control)).

### 2.6. Measurement of Nitric Oxide Production

Following preincubation of Raw 264.7 cells (5 × 10^5^ cells/mL) for 16 h, cells were pretreated with a variety of concentrations of BPTS for 1 h, followed by stimulation with 1 *μ*g/mL of LPS. Cells were then incubated for 20 h at 37°C, 5% CO_2_ incubator, followed by collection of culture supernatants. Nitric oxide was measured by reaction with 100 *μ*L of Griess reagent (1% sulfanilamide and 0.1% N-[1-naphthy]-ethylenediamine dihydrochloride in 5% phosphoric acid; Roche) to 100 *μ*L of culture supernatant for 15 min at room temperature in the dark. Absorbance was determined at 540 nm using an ELISA microplate reader (Tecan, USA). A standard curve was generated in the same fashion using NaNO_2_.

### 2.7. PGE_2_, TNF-*α*, and IL-6 Assays

Raw 264.7 cells (5 × 10^5^ cells/mL) were preincubated for 16 h. Cells were then pretreated with a variety of concentrations of BPTS for 1 h, followed by stimulation with 1 *μ*g/mL of LPS. Following collection of culture supernatants at 20 h after LPS stimulation, levels of PGE_2_, TNF-*α*, and IL-6 were quantified by enzyme-linked immunosorbent assay (ELISA), according to the manufacturer's protocol (PGE_2_, R&D Systems, Minneapolis, MN, USA; TNF-*α* and IL-6, Pierce Biotechnology, Rockford, IL, USA).

### 2.8. Western Blot Analysis

Control and BPTS-treated Raw 264.7 cells were collected by centrifugation and washed once with phosphate-buffered saline (PBS). Washed cell pellets were resuspended in extraction lysis buffer (50 mM HEPES (pH 7.0), 250 mM NaCl, 5 mM EDTA, 0.1% Nonidet P-40, 1 mM PMSF, 0.5 mM DTT, 5 mM NaF, and 0.5 mM sodium orthovanadate) containing 5 *μ*g/mL each of leupeptin and aprotinin and incubated for 20 min at 4°C. Cell debris was removed by microcentrifugation, and supernatants were frozen rapidly. Bio-Rad protein assay reagent was used for determination of protein concentrations, according to the manufacture's instruction. Thirty micrograms of cellular proteins from treated or untreated cell extracts were separated on 8% SDS-polyacrylamide gels and electroblotted onto nitrocellulose membranes, followed by incubation overnight with blocking solution (5% skim milk) at 4°C, and then with primary antibody for 2 h. Blots were then washed three times with Tween 20/Tris-buffered saline (TTBS), incubated with a 1 : 1000 dilution of horseradish peroxidase-conjugated secondary antibody for 1 h at room temperature, and rewashed three times with TTBS. ECL Western detection reagents (Amersham Bioscience, Piscataway, NJ, USA) were used for development of blots.

### 2.9. Preparation of Nuclear Extracts

Dishes were washed with ice-cold PBS, scraped, and transferred to microtubes. Cells were allowed to swell by addition of lysis buffer (10 mM HEPES (pH 7.9), 10 mM KCl, 1.5 mM MgCl_2_, 1 mM dithiothreitol, 0.2% NP-40, and protease inhibitor cocktail (Roche Diagnostics, Indianapolis, IN, USA)). Samples were incubated for 10 min on ice and centrifuged for 5 min at 4°C. Pellets containing crude nuclei were resuspended in 50 *μ*L of extraction buffer containing 20 mM HEPES (pH 7.9), 1.5 mM MgCl_2_, 1 mM dithiothreitol, 420 mM NaCl, 20% glycerol, and protease inhibitor cocktail, followed by incubation for 30 min on a shaker at 4°C. Samples were centrifuged at 16,000 × g for 10 min to obtain supernatant containing nuclear extracts.

### 2.10. Reporter Constructs, Transfection, Reporter Cell Line, and Luciferase Assay

Raw 264.7 cells transfected with a NF-*κ*B reporter construct were kindly provided by Professor Myungsoo Joo (Pusan National University, Korea). Transfected cells (5 × 10^5^ cells/mL) were incubated for 16 h with DMEM containing 10% FBS and pretreated with BPTS for 1 h, followed by stimulation with 1 *μ*g/mL LPS for 20 h. After lysis of cells, luciferase activity was determined using the luciferase assay system (Promega, Madison, CA, USA) and a luminometer (Tecan, USA). NF-*κ*B-mediated luciferase activity was normalized according to the amounts of proteins in total cell lysates. The BCA protein assay kit (Pierce, Rockford, IL, USA) was used for determination of the amount of protein.

### 2.11. Carrageenan-Induced Paw Edema

All animal procedures were conducted in accordance with the institutional guidelines for care and use of laboratory animals. Four-week old Sprague-Dawley rats (male, 80–100 g), which were provided from Samtako Co (Osan, Korea), were acclimatized for 1 week. Animals were supplied with filtered pathogen-free air, commercial rat chow (Nestle Purina PetCare Korea Ltd., Seoul, Korea), and water ad libitum, and were maintained at a temperature between 20 and 23°C with 12 h light and dark cycles and relative humidity of 50%. Rats (*N* = 20) were randomly divided into four groups; thus, each group consisted of five animals. Rats received BPTS, dissolved in water, by oral administration at doses of 0.3 and 1.0 g kg^−1^ day^−1^ for four consecutive days. Dexamethasone, an anti-inflammatory drug, was used as a positive control. To induce acute phase inflammation in the paw, rats received subcutaneous injection of a 1% solution of carrageenan dissolved in saline (0.1 mL per animal) into the right hind paw 1 h after treatment with vehicle or BPTS. Paw volumes were measured up to 3 h after injection at intervals of 1 h. Hind paw volume was determined volumetrically by measurement using a plethysmometer (UGO BASILE; Comerio, VA, Italy).

### 2.12. Statistical Analysis

 Results were expressed as mean ± S.D. of triplicate experiments. Differences in mean values between groups were analyzed by one-way analysis of variance (ANOVA) and independent *t*-test. Values of  *P* < 0.05 were regarded as statistically significant differences.

## 3. Results

### 3.1. Analysis of BPTS

**Figure 1:**
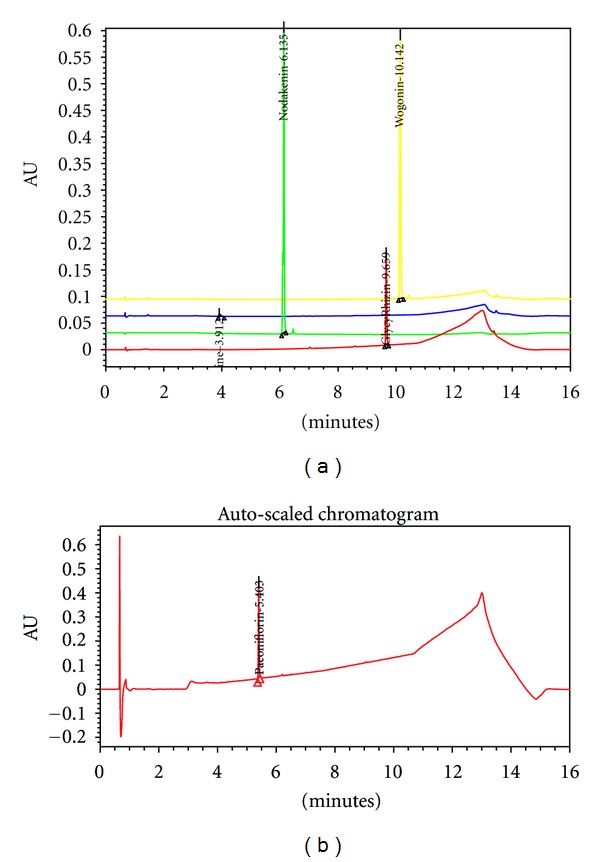
UPLC chromatogram of standard compounds. Standard compounds were subjected to UPLC analysis. The chromatograms were obtained at 230, 254, and 330 nm. Ephedrine (254 nm, 3.912 min), nodakenin (330 nm, 6.135 min), glycyrrhizin (254 nm, 9.659 min), wogonin (254 nm, 10.142 min), (a) and paeoniflorin (230 nm, 5.403 min) (b).

The UPLC system was used in determination of five markers, paeoniflorin, glycyrrhizin, ephedrine, nodakenin, and wogonin, in BPTS. Contents of the five marker components were calculated from the calibration curve of the standards (Table 1 and Figure 1). Validation of the method verified its reliability and stability. Use of the method resulted in successive separation of five marker components in BPTS. 

### 3.2. Effects of BPTS on LPS-Induced Production of NO, PGE_2_, TNF-*α*, and IL-6 and Transcriptional Activity of NF-*κ*B

**Figure 2:**
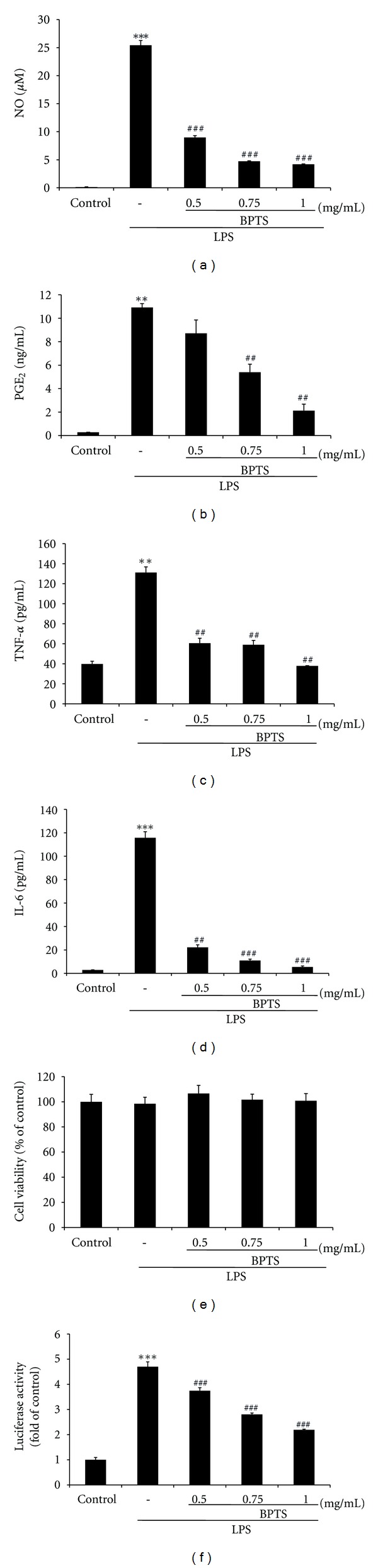
Effects ofBPTS on LPS-induced production of NO (a), PGE_2_ (b) and proinflammatory cytokines (TNF-*α* (c) and IL-6 (d)) in Raw 264.7 macrophages. Cells (5 × 10^5^ cells/mL) were treated with various concentrations (0.5, 0.75, and 1 mg/mL) of BPTS for 1 h, followed by continuous incubation with LPS (1 *μ*g/mL) for the next 20 h. Concentrations of NO, PGE_2_, and proinflammatory cytokines in culture medium were monitored as described in the methods section. Viability of cells exposed to BPTS was measured using the MTT assay (e). Effects of BPTS on LPS-induced NF-*κ*B-dependent luciferase activity (f). Transfected cells (5 × 10^5^ cells/mL) were incubated for 16 h and pretreated with different concentrations (0.5, 0.75, and 1 mg/mL) of BPTS for 1 h, followed by stimulation with 1 *μ*g/mL LPS for 20 h. Following lysis of cells, luciferase activity was determined using the Promega luciferase assay system and a luminometer. Data represent the mean ± S.D. from three separate experiments. **P < 0.01, ***P < 0.001, significant compared with vehicle-treated control; ^##^ *P <* 0.01, ^###^ *P* < 0.001, significant compared with LPS alone.

NO and PGE_2_ are produced by immune-activated macrophages, and at inflammatory sites [29]. To determine the effects of BPTS on LPS-induced NO and PGE_2_ production in Raw 264.7 cells, different dosages of BPTS (0.5, 0.75, and 1 mg/mL) were chosen. After treatment with LPS, NO concentration showed an increase of approximately 195.4-fold (25.4 ± 0.84 *μ*M), compared to the control (0.13 ± 0.04 *μ*M). Treatment with BPTS resulted in inhibition of LPS-induced NO production in a dose-dependent manner: 65%, 82%, and 84% at dosages of 0.5, 0.75, and 1 mg/mL, respectively (Figure 2(a)). In addition, treatment with LPS resulted in an increase in PGE_2_ concentration by approximately 38.6-fold (10.9 ± 0.3 ng/mL), compared to the control (0.282 ± 0.001 ng/mL). Treatment with BPTS resulted in significant inhibition of LPS-induced production of PGE_2_ in a dose-dependent manner: 52% and 83% at dosages of 0.75 and 1 mg/mL, respectively (Figure 2(b)). Next, we evaluated the effects of BPTS on the LPS-induced production of TNF-*α* and IL-6 by enzyme immunoassay. Compared to control, treatment with LPS resulted in significantly increased production of proinflammatory cytokines (TNF-*α*, 3.3-fold; IL-6, 41.0-fold) in culture supernatants of Raw 264.7 cells. Treatment with BPTS at concentrations of 0.5, 0.75, and 1 mg/mL resulted in significant inhibition of LPS-induced production of TNF-*α* and IL-6 (Figures 2(c) and 2(d)), suggesting that BPTS inhibits expression of these particular genes involved in the inflammation process. These results indicate that BPTS is a strong inhibitor of TNF-*α* and IL-6 production. In addition, an MTT assay was used for examination of possible cytotoxic effects of BPTS in Raw 264.7 cells. Results showing that cell viability was not affected by treatment with BPTS, at least up to the BPTS concentration of 1 mg/mL (Figure 2(e)), demonstrated that no cytotoxic effect of BPTS was observed. In addition, we examined the effects of BPTS on induction of NF-*κ*B transcriptional activity by LPS. Transfected cells were stimulated with 1 *μ*g/mL LPS either in the presence or absence of BPTS. Treatment with BPTS at 0.5, 0.75, or 1 mg/mL resulted in significant reduction of LPS-induced increases in NF-*κ*B dependent luciferase activity (Figure 2(f)).

### 3.3. Effects of BPTS on LPS-Induced Expression of iNOS and COX-2

**Figure 3:**
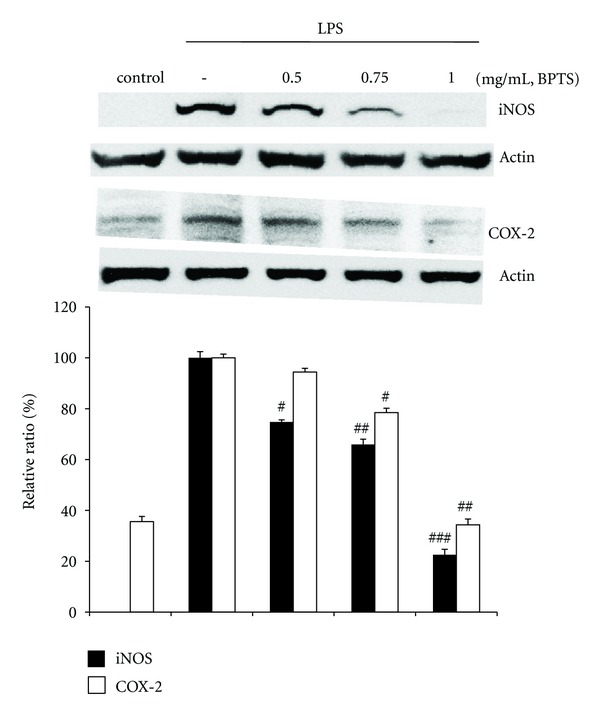
Effects of BPTS on LPS-induced expression of iNOS and COX-2. Raw 264.7 cells (5 × 10^5^ cells/mL) were treated with various concentrations (0.5, 0.75, and 1 mg/mL) of BPTS for 1 h, followed by continuous incubation with LPS (1 *μ*g/mL) for the next 20 h. Control cells were incubated with vehicle alone. Western blot analysis was performed for determination of protein levels of iNOS and COX-2. *β*-Actin was used as a loading control. The blots shown are representative of three blots yielding similar results. iNOS and COX-2 versus *β*-actin were measured via densitometry. Data represent the mean ± S.D. from three separate experiments. ^#^ *P* < 0.05, ^##^ *P* < 0.01, and ^###^ *P* < 0.001 indicate significant differences from the LPS-induced group.

To investigate whether the inhibitory effects of BPTS against NO and PGE_2_ production were related to iNOS and COX-2 modulation, western blot analysis was performed. As shown in Figure 3, iNOS and COX-2 protein levels were outstandingly upregulated in response to LPS. Treatment with BPTS resulted in dose-dependent inhibition of LPS-induced iNOS and COX-2 protein levels. In particular, treatment with BPTS (1 mg/mL) resulted in powerful inhibition of iNOS and COX-2 protein levels. These results were consistent with the inhibitory effects of BPTS on production of NO and PGE_2_.

### 3.4. Effects of BPTS on LPS-Induced Activation of NF-*κ*B

**Figure 4:**
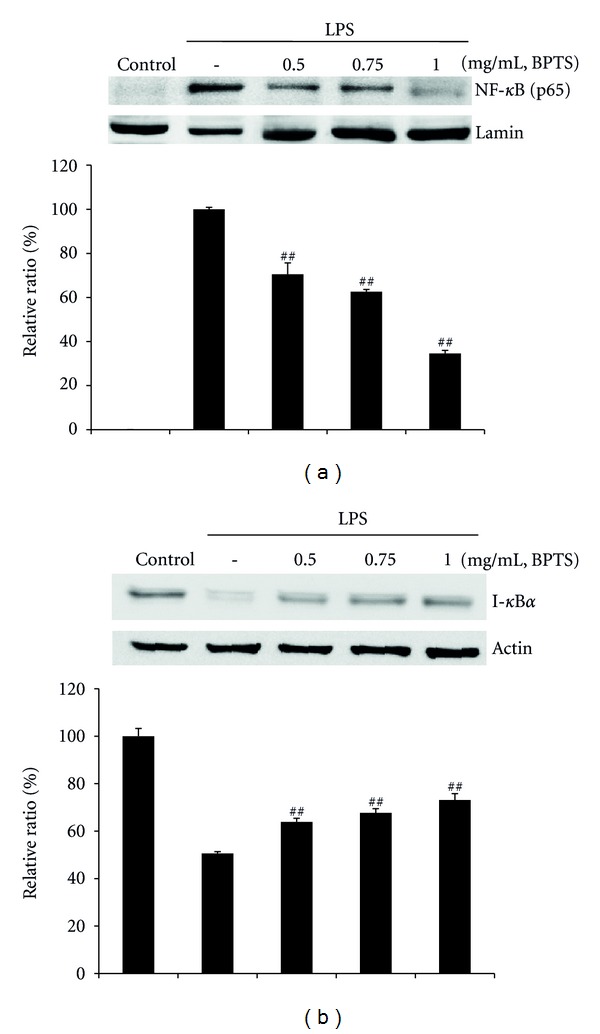
Effects of BPTS on LPS-induced activation of NF-*κ*B (p65) and degradation of I-*κ*B*α*. Cells (5 × 10^5^ cells/mL) were treated with BPTS (0.5–1 mg/mL) for 1 h, followed by continuous incubation with LPS (1 *μ*g/mL) for 15 min. Control cells were incubated with vehicle alone. Nuclear extracts for NF-*κ*B were prepared as described in the methods section. Western blot analysis was performed for determination of protein levels of NF-*κ*B (p65 subunit) (a) and I-*κ*B*α* (b). The blots shown are representative of three blots yielding similar results. NF-*κ*B (p65) versus Lamin A/C (a) and I-*κ*B*α* versus *β*-actin (b) were measured via densitometry. Data represent the mean ± S.D. from three separate experiments. ^##^ *P* < 0.01 and ^###^ *P* < 0.001 indicate significant differences from the LPS-induced group.

NF-*κ*B regulates both innate and adaptive immune responses. It is activated rapidly in response to a wide range of stimuli, including pathogens, stress signals, and proinflammatory cytokines, such as LPS and TNF [30]. In order to evaluate the question of whether inhibition of nuclear translocation of NF-*κ*B by BPTS is due to suppression of I-*κ*B*α* degradation, western blot analysis was performed for immunological determination of the levels of NF-*κ*B (p65) and I-*κ*B*α*. Findings from studies by Kim et al. [31] demonstrated that activation of NF-*κ*B occurred 30 min to 1 h after treatment with LPS. However, in our study, activation of NF-*κ*B occurred 15 min after treatment with LPS and strong inhibition of NF-*κ*B activation by BPTS was observed at a concentration of 1 mg/mL (Figure 4(a)). Nuclear translocation of NF-*κ*B is preceded by degradation of the I-*κ*B*α* subunit. We found that addition of LPS led to a reduction of I-*κ*B*α* level at 15 min, and treatment with BPTS resulted in blockade of LPS-induced I-*κ*B*α* degradation (Figure 4(b)). These results were consistent with Kim's observation [32] that exposure to LPS resulted in decreased levels of I-*κ*B*α* protein 15 min after treatment, and treatment with liquiritigenin allowed cells to recover the level of I-*κ*B*α*. Our results demonstrated that treatment with LPS resulted in degradation of I-*κ*B*α*, whereas treatment with BPTS resulted in inhibited nuclear translocation of NF-*κ*B and degradation of I-*κ*B*α*. Consistent with these results, a dose-dependent reduction of transcriptional activity of NF-*κ*B was also observed after pretreatment with BPTS.

### 3.5. Effects of BPTS on LPS-Induced Phosphorylation of MAPKs

**Figure 5:**
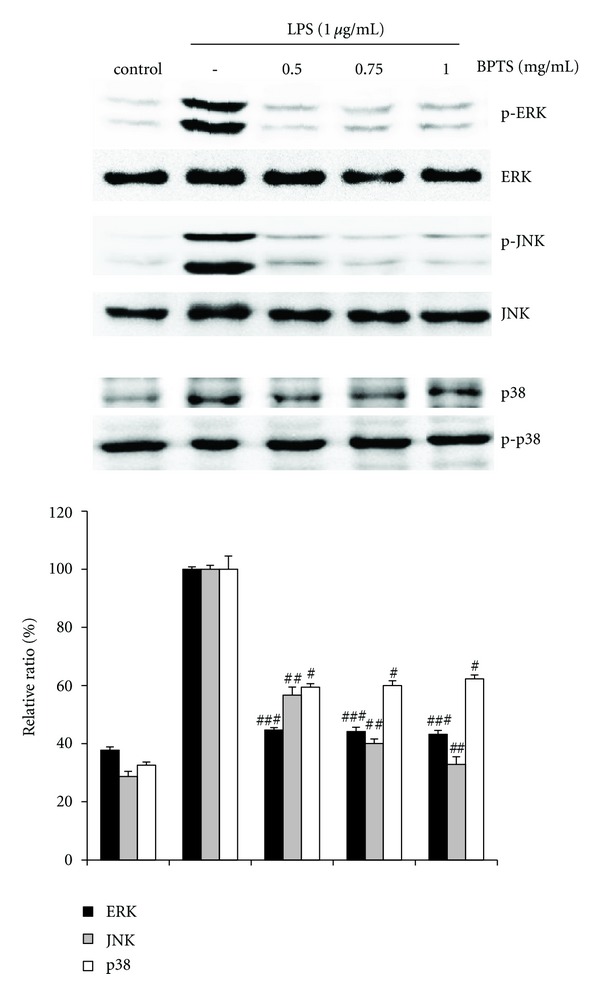
Effects of BPTS on the phosphorylation of MAPKs (ERK, JNK, and p38) in the LPS-induced Raw 264.7 cells. Cells were treated with indicated concentrations of BPTS for 1 h and induced with 1 *μ*g/mL LPS for 15 min. Equal amounts of cell extracts were analyzed by western blot analysis with antiphosphokinase antibodies, respectively. Western blot detection of nonphosphorylated kinases was estimated with the protein loading control for each lane. The blots shown are representative of three blots yielding similar results. Data represent the mean ± S.D. from three separate experiments. ^##^ *P* < 0.01 and ^###^ *P* < 0.001 indicate significant differences from the LPS-induced group.

To investigate the molecular target of BPTS in the further upstream signaling pathway, we evaluated the effects of BPTS on the LPS-induced phosphorylation of ERK1/2, JNK, and p38 MAPKs in Raw 264.7 cells. As shown in Figure 5, treatment with LPS (1 *μ*g/mL) for 15 min resulted in significantly increased phosphorylation of ERK1/2, JNK, and p38 MAPKs. Treatment with BPTS (0.5, 0.75, and 1 mg/mL) resulted in significantly reduced phosphorylation of ERK1/2, JNK, and p38 MAPKs. However, the amounts of nonphosphorylated ERK1/2, JNK, and p38 MAPKs were unaffected by treatment with either LPS or BPTS.

### 3.6. Inhibitory Effects of BPTS on Carrageenan-Induced Edema in Rats

**Figure 6:**
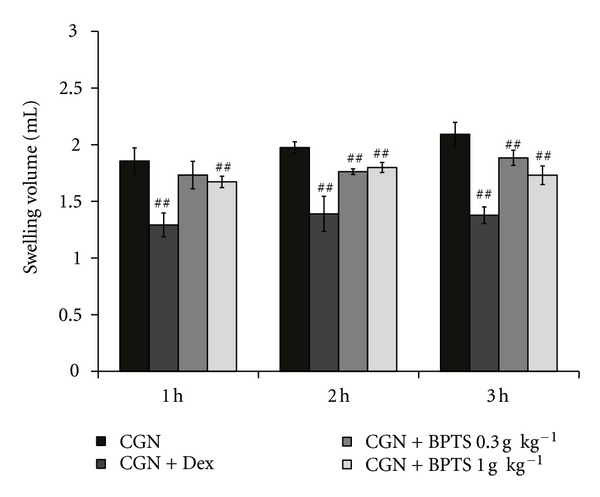
Inhibition of carrageenan- (CGN-) induced paw edema by BPTS. BPTS was administered to rats at an oral dose of 0.3 and 1.0 g kg^−1^ day^−1^ for four days prior to induction of paw edema. Paw edema was induced by subcutaneously injecting a 1% solution of carrageenan dissolved in saline (0.1 mL per animal) into the right hind paw. The swelling volume of the paw was measured 1–3 h after carrageenan injection. Dexamethasone (Dex, 1 mg kg^−1^, p.o.) was used as a positive control. Data represent the mean ± S.D. of five animals. ^##^ *P* < 0.01, significant compared with carrageenan alone.

We performed a paw edema assay to assess whether BPTS affected acute phase inflammation in an animal model, because BPTS effectively inhibited iNOS, COX-2 and proinflammatory cytokines inductions in macrophages. In this study, treatment of rats with BPTS at an oral dose of 1 g kg^−1^ day^−1^ for four days resulted in significantly (*P* < 0.01) reduced paw swelling at 1, 2, and 3 h, in comparison with the carrageenan control. Treatment with dexamethasone (a positive control) also resulted in significantly (*P* < 0.01) decreased edema formation (Figure 6). 

## 4. Discussion

Inflammation is a protective host response to pathogenic stimuli and is characterized by the cardinal signs such as heat, swelling, redness, and pain. In oriental medicine, effective therapeutic results have been achieved by treatment of pathogenic heat with BPTS [2]. However, there has been little scientific evidence to prove the effectiveness of BPTS. Therefore, in this study, we investigated its anti-inflammatory effects. 

Proinflammatory mediators NO and PGE_2_ are generated by iNOS and COX-2 during the inflammatory response [33–35]. Tomé et al. [36] demonstrated previously that iNOS causes damage to normal cells through production of a large amount of NO in macrophages treated with LPS. In addition, it has been reported that during the inflammatory response COX-2 is activated to produce PGE_2_ which contributes to formation of tumors through inhibition of apoptosis and induction of cell division, cancer metastasis, and angiogenesis [37]. Thus, inhibitors of these inflammatory mediators have been regarded as potential candidates for use as anti-inflammatory agents. In this study, we demonstrated that treatment with BPTS resulted in effective inhibition of LPS-induced NO production via suppressing expression of iNOS protein. Similar to NO, we found that BPTS also inhibited PGE_2_ production via suppressing expression of COX-2 protein in LPS-stimulated macrophages. From these results, we suggest that the therapeutic effect of BPTS in treatment of pathogenic heat is due in part to its inhibition of NO and PGE_2_ production.

TNF-*α* and IL-6 are frequently encountered proinflammatory cytokines, involved in regulation and mediation of immunity and inflammation. Involvement of these cytokines in a variety of immunological functions as well as interaction with a variety of target cells has been reported [11–13]. Release of large amounts of TNF and IL-6 in response to LPS in macrophages has been reported [13]. In addition, induction of these cytokines was found to be dependent on NF-*κ*B activation [38]. In the present study, we demonstrated that treatment with BPTS resulted in significantly inhibited secretion of LPS-induced TNF-*α* and IL-6 in macrophages. These findings indicate that the inhibitory effects of BPTS on proinflammatory cytokines has important implications for development of anti-inflammatory agents from natural herbs.

NF-*κ*B is a ubiquitous transcription factor that controls expression of genes involved in immune responses, apoptosis, and the cell cycle. Inappropriate activation of NF-*κ*B can mediate inflammation and tumorigenesis. In addition, development of a drug that can control NF-*κ*B is a strategy for treatment of inflammatory diseases [39]. Exposure of cells to LPS leads to phosphorylation and degradation of I-*κ*B proteins, followed by translocation of NF-*κ*B to the nucleus. In the nucleus, NF-*κ*B dimers combine with target DNA elements to activate transcription of genes encoding for proteins involved in inflammation [30, 40]. Our data showed that treatment with LPS resulted in degradation of I-*κ*B*α*, whereas treatment with BPTS resulted in inhibited nuclear translocation of NF-*κ*B and degradation of I-*κ*B*α*. These findings indicate that BPTS may inhibit NF-*κ*B activation by suppressing degradation of I-*κ*B*α* and nuclear translocation of NF-*κ*B in LPS-stimulated macrophages. In addition, treatment with BPTS reduced LPS-induced increases in NF-*κ*B dependent luciferase activity. This result is associated with inhibition of nuclear translocation of NF-*κ*B and degradation of I-*κ*B*α* by BPTS. In addition, involvement of NF-*κ*B in regulation of COX-2 and iNOS expression, as well as inhibited expression of COX-2 and iNOS through blockade of improper NF-*κ*B activation by several chemopreventive phytochemicals has been reported [29, 32]. In addition, the NF-*κ*B transcription pathway is a key regulator of LPS-stimulated release of proinflammatory cytokines [41]. Results of the present study indicate that the inhibitory activity of BPTS against NF-*κ*B activation stimulated by LPS shows correlation with inhibition of proinflammatory mediators, including NO, PGE_2_, TNF-*α*, and IL-6.

MAPKs (ERK1/2, JNK, and p38) are a family of protein serine/threonine kinases that are an important part of intracellular signaling pathways [35, 42, 43]. LPS induction of murine macrophages, leading to increased phosphorylation and activation of ERK1/2, JNK, and p38 MAPKs has been reported [44]. Both the ERK and p38 pathways play a role in upregulation of iNOS and production of TNF during macrophage activation [23, 45]. In addition, the JNK signaling cascade regulates expression of LPS-stimulated iNOS [46]. Berghe et al. [47] reported on repression of nuclear NF-*κ*B activity and transactivation activity of NF-*κ*B (p65) by specific inhibitors of the ERK and p38 MAP kinase pathways. In this study, we demonstrated that treatment with BPTS significantly inhibited phosphorylation of MAPKs. In addition, results of the present study demonstrate the association of inhibited phosphorylation of MAPKs with NF-*κ*B inactivation. Thus, our findings suggest that the anti-inflammatory effects of BPTS are due to inhibition of LPS-induced NF-*κ*B activation and phosphorylation of MAPKs in Raw 264.7 cells.

To investigate the anti-inflammatory effects of BPTS *in vivo*, we performed a carrageenan-induced paw edema assay in an animal model. Carrageenan-induced paw edema is widely employed for screening the effects of anti-inflammatory drugs [48]. Intraplantar injection of carrageenan induces inflammatory responses, including increases in capillary permeability, leukocyte infiltration, migration of neutrophils and macrophages, paw edema, and development of hyperalgesia [49, 50]. Several other research groups have reported that carrageenan induces peripheral release of NO in the later stage of carrageenan-induced inflammation [51] as well as that of prostaglandin E_2_ and proinflammatory cytokines such as TNF-*α* and IL-6 [52, 53]. This carrageenan-induced paw edema assay enabled us to demonstrate the effects of BPTS in inhibition of swelling induced by acute inflammation. Our results suggest that inhibition of LPS-induced NO, PGE2, TNF-*α*, and IL-6 production by BPTS in macrophages may represent crucial mechanisms involved in inhibition of carrageenan-induced formation of paw edema. 

The BPTS prescription is comprised of 17 medical herbs and talcum. Several herbal constituents of BPTS have been reported to exert anti-inflammatory effects, as follows. *Ephedrae herba* has been reported to decrease LPS-induced COX-2 expression and NF-*κ*B dependent transcription in C6 rat glioma cells [54]. Ephedrine, a primary component of *Ephedrae herba*, is used for treatment of rhinitis as well as acute asthma attacks, bronchial asthma, and allergy [55, 56]. The ethylacetate fraction of *Ledebouriellae radix* can induce significant suppression of Freund's complete adjuvant-induced paw edema as well as the serum level of IL-6 [57]. Paeoniflorin, a component of *Paeoniae radix*, has been reported to inhibit NF-*κ*B expression in chronic hypoperfusion rats and to have anti-inflammatory properties [58]. Findings from a recent study demonstrated that glycyrrhizin, a triterpene glycoside from licorice root (*Glycyrrhizae radix*), causes broadly inhibited induction of proinflammatory mediators induced by TLR 9 agonist CpG-DNA in Raw 264.7 cells, and exerts strong attenuation of inflammatory responses induced by TLR 3 and TLR 4 ligands [59]. In addition, inhibition of interleukin-8 production and NF-*κ*B activity in lung epithelial cells and attenuated development of carrageenan-induced lung injury in mice by treatment with glycyrrhizin have been reported [60, 61]. Nodakenin, a coumarin compound from the roots of *Angelica gigas*, has anodyne activities [62] and antiallergic effects in IgE/Ag-induced type I hypersensitivity [63]. Finally, wogonin (5,7-dihydroxy-8-methoxyflavone), a flavone from *Scutellariae radix*, has been reported to suppress COX-2 expression in skin fibroblasts [64]. It is suggested that the anti-inflammatory effects of BPTS, as observed in murine macrophages stimulated by LPS and carrageenan-induced paw edema in our experiment, are probably due to these components. However, given that the constituents of other herbal components remain unclear, important roles for other components cannot be ruled out. Using the HPLC system, Weon et al. [65] reported on determination of five main markers, including paeoniflorin, glycyrrhizin, 6-gingerol, decursin, and geniposide in BPTS. However, according to our analytical results, main markers of BPTS included paeoniflorin, glycyrrhizin, ephedrine, nodakenin, and wogonin. Marker components can be affected by many factors, including cultivation environment or collection time of herbs and method of manufacture of the herbal formula. For this reason, the types of marker compounds determined in the two groups are thought to be different. 

**Figure 7:**
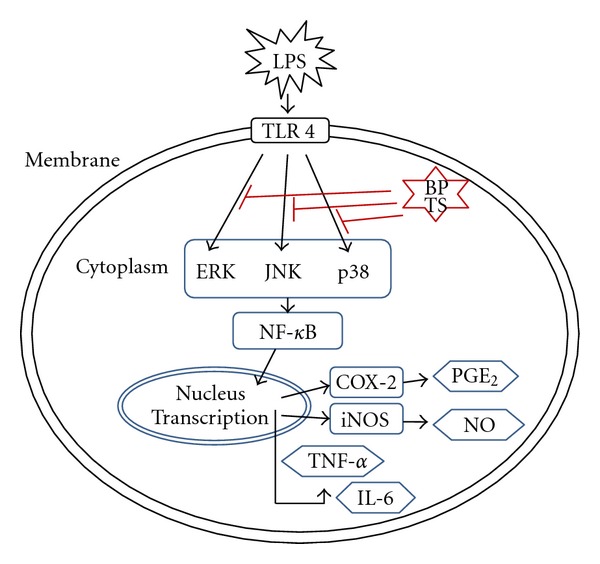
Proposed mechanisms for anti-inflammatory effects of BPTS in LPS-induced macrophages.

In conclusion, our results suggest that BPTS may exert anti-inflammatory effects via reduction of proinflammatory mediators, including NO, PGE_2_, TNF-*α*, and IL-6 through suppression of the signaling pathways of NF-*κ*B and MAPKs in LPS-induced macrophages (Figure 7). In addition, BPTS was found to have an antiedema effect in the carrageenan-induced paw edema assay in rats. These findings may provide scientific evidence validating the use of BPTS in treatment of patients with heat syndrome in Korean oriental medicine. 

## Figures and Tables

**Table 1:** Contents of five marker components of BPTS by UPLC (*n* = 3).

Compound	Content (*μ*g/mL)
Paeoniflorin	134.3 ± 0.8
Glycyrrhizin	234.7 ± 3.03
Ephedrine	534.9 ± 3.0
Nodakenin	39 ± 0.1
Wogonin	15 ± 1
